# Radiation-Induced Tumor Lysis Syndrome in Chronic Lymphocytic Leukemia

**DOI:** 10.4274/tjh.2015.0259

**Published:** 2016-08-19

**Authors:** Ali Alkan, Tuğçe Kütük, Ebru Karcı, Arzu Yaşar, Ayşe Hiçsönmez, Güngör Utkan

**Affiliations:** 1 Ankara University Faculty of Medicine, Department of Medical Oncology, Ankara, Turkey; 2 Ankara University Faculty of Medicine, Department of Radiation Oncology, Ankara, Turkey

**Keywords:** Chronic lymphocytic leukemia, Radiation, tumor lysis syndrome

## Abstract

Tumor lysis syndrome (TLS) is an important oncological emergency that is usually observed with hematological malignancies and rarely with solid tumors. It can be induced either by therapy or spontaneously. Radiotherapy-induced TLS has been rarely reported in the literature. Here we present a patient with a diagnosis of metastatic prostate cancer and chronic lymphocytic leukemia complicated with TLS during palliative radiotherapy.

## Abstract (tr)

Tümör lizis sendromu (TLS) sıklıkla hematolojik malignitelerde görülen, nadiren solid tümörlerde karşımıza çıkabilen bir onkolojik acildir. Tedavi ilişkili olabileceği gibi spontan olarak da ortaya çıkabilir. Radyasyon ilişkili TLS literatürde nadiren bildirilmiştir. Burada, kronik lenfositik lösemi ve prostat kanseri tanısıyla izlenen hastada palyatif radyoterapi ile indüklenen TLS olgusu sunulmaktadır.

## INTRODUCTION

Tumor lysis syndrome (TLS) is one of the important oncological emergencies in oncology practice, especially in lymphoproliferative malignancies. Aggressive solid tumors with shorter doubling time can be complicated with TLS. Although it is generally triggered by cytotoxic therapy, it can also be observed spontaneously in cases of bulky tumors. Radiation as a cause of TLS has been rarely reported in the literature [1,2,3]. Here we present a patient with 2 primary malignancies experiencing TLS during palliative radiotherapy.

## CASE PRESENTATION

A 69-year-old male patient, without any comorbidities, presented with fullness in the left axilla. The initial examination showed lymphocytosis and lymphadenopathies in the bilateral axillary and inguinal region. There was neither hepatosplenomegaly nor pathological lymph nodes in the abdominal and thoracic cavity. Lymphocytosis and basket cells were seen in the blood smear. Excisional biopsy from the left axilla showed infiltration by CD5-positive cells. Immunohistochemistry was consistent with a diagnosis of chronic lymphocytic leukemia (CLL) infiltration. The bone marrow biopsies supported the diagnosis with hypercellularity and diffuse interstitial infiltration of small atypical lymphoid cells. The patient had been followed with a diagnosis of CLL for 2 years. Due to initial stage 1 disease, the patient had been periodically followed without medical therapy.

**Figure 1:**
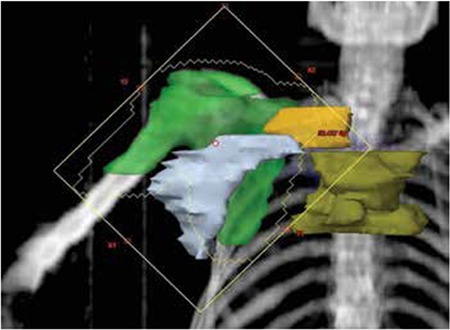
Digitally reconstructed radiograph of the scapula field including axillary lymph nodes, supraclavicular lymph nodes, and the upper mediastinal lymph node area.

In a routine outpatient visit, the clinically asymptomatic patient was evaluated with lymph node excisional biopsy due to progression of the pathological lymph nodes in order to exclude Richter’s transformation. In addition, bone marrow sampling was performed. Pathology revealed small atypical lymphoid cells consistent with an ongoing lymphoproliferative disease and infiltration of an adenocarcinoma as a second primary malignancy. Further immunohistochemical staining was inconclusive for the origin of the tumor. The age of the patient, osteoblastic metastatic lesions detected in a bone scan, and prostate-specific antigen (PSA) level of 1712 ng/mL led us to order a work-up for prostate cancer. Transrectal prostate biopsy showed prostate adenocarcinoma with a Gleason score of 10. The patient was treated with goserelin (10.8 mg SC, every 12 weeks) and bicalutamide (50 mg daily). The analysis of bone scans before hormonal therapy showed metastatic lesions in the bilateral scapula, thoracic vertebral column, pelvis, bilateral humerus, and femur. For skeletal metastatic disease, zoledronic acid (4 mg intravenous, every 4 weeks) was started and palliative radiotherapy was planned for painful metastatic lesions in the thoracic vertebrae. Radiotherapy to T3-6 and the right scapula with a total of 30 Gy divided into 10 fractions was planned. Pathological lymph nodes associated with CLL, ranging between 1 and 3 cm in diameter in the bilateral inguinal areas, axilla, neck, and hilum, were noted for follow-up. On day 7 of radiotherapy the patient complained about mild nausea, progressive malaise, and perioral numbness. Physical examination was normal except for pathological lymph nodes with minimal regression and paleness. The largest lymph node in the right axilla had regressed from 3 to 2 cm and the other pathological nodes were stable. The hilar fullness in chest X-ray was stable. The only medications used were drugs for prostate cancer (bicalutamide and goserelin), lansoprazole for dyspepsia, and dexamethasone (8 mg), which was planned with the radiotherapy. The laboratory evaluation revealed acute renal failure complicated with TLS (Table 1). The PSA value was stable. The patient was hospitalized and aggressively hydrated with 0.9% NaCl at 500 mL/h in the first 6 h of follow-up with monitorization of hourly urine output. In the initial evaluation, due to hypocalcemia (5.2 mg/dL) with neuromuscular symptoms and prolonged QT interval of 0.50 s, the patient was treated with calcium gluconate replacement under cardiac monitorization. Laboratory results were checked at 4-h intervals. Until we could use rasburicase, allopurinol was the initial specific therapy for TLS. The dosage was titrated according to glomerular filtration rate and a 0.2 mg/kg single dose of rasburicase could be added to therapy on day 3 of hospitalization. The patient’s symptoms and renal dysfunction progressively improved (Table 1). The patient was free of any findings of infection or septicemia. The leukocytosis was linked to the dexamethasone therapy, which was planned during radiotherapy for anti-edema prophylaxis. After stopping the drug, leukocytosis improved. Analysis of the dose-volume histogram showed that during palliative radiotherapy to the thoracal vertebrae, mediastinal, axillary, and supraclavicular lymph nodes were also affected with maximums of 24.8 Gy (mean: 19.8), 34.2 Gy (mean: 20), and 28.1 Gy (mean: 19.1), respectively. A digitally reconstructed radiograph of the scapula field included axillary lymph nodes, supraclavicular lymph nodes, and the upper mediastinal lymph node area (Figure 1). After improvement of TLS, reanalysis of the patient for progression of CLL showed minimal regression of the pathological mediastinal and axillary lymph nodes. There was no progression in other lymph nodes or new organomegaly. With stable clinical findings of CLL and history of lymph node resection three months ago without any aggressive form of lymphoproliferative disease or Richter transformation, rebiopsy was not planned. After six months of follow-up the patient was stable for CLL and the PSA level progressively decreased.

## DISCUSSION AND REVIEW OF THE LITERATURE

TLS is an oncological emergency that results from massive tumor lysis due to therapy or spontaneous bursting of tumor cells. The release of intracellular electrolytes into circulation causes an electrolyte imbalance and, as a result, acute renal failure. Intracellular nucleic acid catabolism and as a result hyperuricemia further contribute to renal dysfunction. The clinical presentation may range from nonspecific malaise and nausea to sudden cardiac death. TLS is diagnosed by both laboratory and clinical findings. Laboratory TLS is defined as the imbalance of two or more electrolytes (hypocalcemia, hyperuricemia, hyperphosphatemia, hyperkalemia), whereas clinical TLS is defined as laboratory TLS plus one clinical finding (increased creatinine, arrhythmia/sudden death, seizure) [4,5,6,7].

In the literature, radiation has been rarely reported as a cause of TLS. Most reported cases are associated with hematological pathologies and splenic radiation is the most often related scenario [8]. Total body irradiation before allogeneic stem cell transplantation has been related to TLS [9]. TLS as a complication of radiation in solid tumors has been rarely reported. Palliation of bone metastasis from malignant melanoma [10] and prostate carcinoma [3] has been discussed. The outcomes of the patients were good and there were no mortalities as a result of complications.

After the diagnosis of a second primary tumor, palliative radiotherapy was indicated due to bone pain refractory to analgesia. The radiotherapy plan for the right scapula and thoracal vertebrae involved the right axilla and mediastinum incidentally, where pathological lymph nodes had been documented. The regression of axillary pathological lymph nodes and stable PSA values after radiotherapy led us to a link between TLS and the radiated pathological lymph nodes. CLL with a leukocyte value of <50,000/µL is accepted as low risk for TLS and there is no recommended precaution except for hydration [11]. Due to the absence of any therapy for CLL, there was no precaution for TLS.

Similar to the cases in the literature, our patient improved progressively with supportive care. To the best of our knowledge, our experience is the first reported TLS case related to radiation in a CLL patient. While our case gives important clues about a rare complication of radiation, it also reminds us of the importance of radiation planning in a patient with lymphadenopathies.

## Ethics

Informed Consent: It was taken.

## Figures and Tables

**Table 1:**
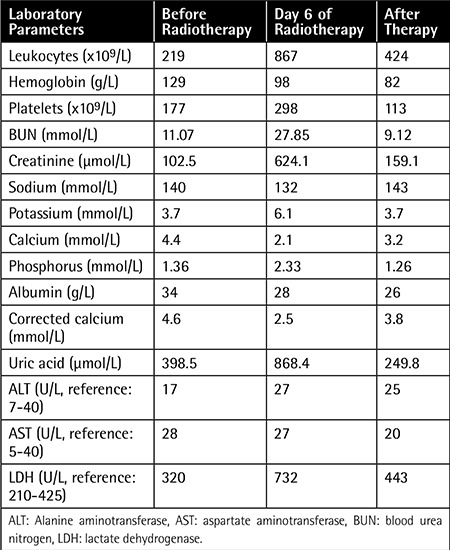
The laboratory parameters before and after radiotherapy and after treatment.
